# *DUX4* Expression in FSHD Muscles: Focus on Its mRNA Regulation

**DOI:** 10.3390/jpm10030073

**Published:** 2020-07-28

**Authors:** Eva Sidlauskaite, Laura Le Gall, Virginie Mariot, Julie Dumonceaux

**Affiliations:** 1 NIHR Biomedical Research Centre, University College London, Great Ormond Street Institute of Child Health and Great Ormond Street Hospital NHS Trust, London WC1N 1EH, UK; e.sidlauskaite@ucl.ac.uk (E.S.); l.gall@ucl.ac.uk (L.L.G.); virginie.mariot@ucl.ac.uk (V.M.); 2 Northern Ireland Center for Stratified/Personalised Medicine, Biomedical Sciences Research Institute, Ulster University, Derry~Londonderry, Northern Ireland BT47 6SB, UK

**Keywords:** FSHD, DUX4, transcription, muscle, regulation

## Abstract

Facioscapulohumeral dystrophy (FSHD) is the most frequent muscular disease in adults. FSHD is characterized by a weakness and atrophy of a specific set of muscles located in the face, the shoulder, and the upper arms. FSHD patients may present different genetic defects, but they all present epigenetic alterations of the D4Z4 array located on the subtelomeric part of chromosome 4, leading to chromatin relaxation and, ultimately, to the aberrant expression of one gene called *DUX4*. Once expressed, DUX4 triggers a cascade of deleterious events, eventually leading to muscle dysfunction and cell death. Here, we review studies on *DUX4* expression in skeletal muscle to determine the genetic/epigenetic factors and regulatory proteins governing *DUX4* expression, with particular attention to the different transcripts and their very low expression in muscle.

## 1. Introduction

Double homeobox 4 (*DUX4*) is a transcription factor that is normally expressed during embryonic development and in the human testes but suppressed in somatic tissue (for review see [1]). The recent finding of DUX4 in an early cleavage-stage embryo raised the hypothesis that DUX4 might act as a functional transcriptional programmer to activate the cleavage-stage transcriptional platform and might be a key regulator of zygotic genome activation [2,3,4]. Moreover, the presence of DUX4 in the testis suggests that *DUX4* may be activated in the primary spermatocytes during spermatogenesis [5]. More recently, *DUX4* activation gained a particular interest across cancer research, as *DUX4* expression in tumours results in immune evasion [6].

Despite the awareness of *DUX4* expression in normal germline biology, DUX4 is principally described as a toxic factor involved in facioscapulohumeral dystrophy (FSHD) pathophysiology. Indeed, in FSHD patients, DUX4 is aberrantly expressed in the muscle tissue [5,7]. The role of DUX4 in FSHD pathogenesis is intensively investigated, and several reviews have been published in this topic [8,9] explaining the potential role of DUX4 in cell death and discussing the role of DNA methylation in FSHD1 and 2 patients. The current review focuses on the recent understanding and regulation of *DUX4* mRNA expression at the mRNA level in skeletal muscle and myogenic cells.

## 2. FSHD

FSHD is the third most common genetic muscular dystrophy with a frequency between 1/8000 to 1/20,000 (www.orpha.net, April 2020). The primary manifestation of FSHD is an asymmetric atrophy of the muscles located in the face, the shoulder, and the upper arm. The pathology often begins during late adolescence; however, the presence of symptoms at an early age is often associated with more severe muscle weakness (reviewed in [10]). The mutation that causes FSHD was identified nearly 30 years ago [11]. FSHD is associated with genetic and epigenetic molecular changes of the D4Z4 microsatellite repeats in the subtelomeric region of chromosome 4 [12,13]. There are two different genetic mechanisms leading to FSHD, and both are associated with the loss of epigenetic marks within the D4Z4 and the aberrant expression of *DUX4* [14]. The first one concerns 95% of FSHD patients (known as FSHD1, OMIM#158900) who show a contraction of a tandemly repeated 3.3 kb microsatellite D4Z4 repeat at the distal end of chromosomal region 4q35. The number of D4Z4 repeats usually varies from 11 to 150, while fewer repeats are observed in less than 3% of the population [15]. In FSHD1 patients, this number is reduced to 10 and below [16]. This reduction of D4Z4 unit number is associated with chromosome relaxation and loss of repression of *DUX4* gene (OMIM#606009), allowing DUX4 transcription in muscle cells [17]. The second one concerns the remaining 5% of FSHD patients (known as FSHD2, OMIM#158901), who do not present a shortened D4Z4 array but carry a mutation in epigenetic modifier genes. The vast majority of FSHD2 cases have been linked to mutations in the *SMCHD1* (structural maintenance of chromosomes flexible hinge domain containing 1) gene [18], encoding a remodelling protein essential for DNA methylation. Few FSHD2 cases present a heterozygous mutation in the *DNMT3B* (DNA methyltransferase 3 beta) gene [19], which is normally responsible for the establishment of the cytosine methylation profile during development. The exact mechanism of how particular mutations cause the FSHD pathology is still under investigation, but the notion of permissive chromosome 4 is now acknowledged for FSHD patients. This “pathological” chromosome 4 is characterized by the following: the presence of specific simple sequence length polymorphism (SSLP) located 3.5 kb proximal to the D4Z4 repeat [20]; the presence of at least one D4Z4 repeat [21]; a chromatin relaxation within the D4Z4 repeat [17]; and the presence of the 4qA haplotype [22,23] containing the polyadenylation signal for DUX4 [14]. Indeed, each D4Z4 contains the open reading frame (ORF) of the *DUX4* retrogene [7,24]. DUX4 protein and mRNA are detected in both FSHD1 and FSHD2 muscle biopsies at very low levels [5] but sufficient to induce a cascade of mis-regulated genes [25] eventually leading to muscle atrophy and muscle fibre death by the disruption of multiple cellular processes (for review see [8]).

## 3. Regulation of *DUX4* Expression

**Figure 1:**
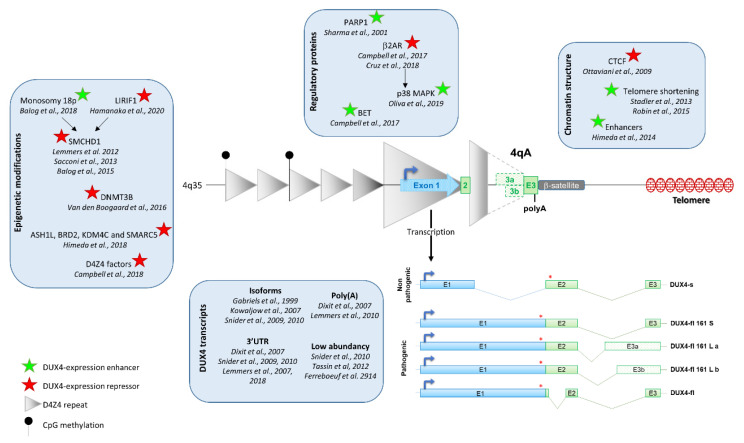
Regulation of *DUX4* expression.

There is a consensus in the scientific community on *DUX4* expression in FSHD biopsies, but its regulation still needs to be deciphered. Indeed, *DUX4* expression is regulated by several factors including D4Z4 epigenetic modification, chromosome conformation and the presence of myogenic enhancers (Figure 1).

*DUX4* expression is regulated by several factors including D4Z4 epigenetic modification, chromatin structure, regulatory proteins, and myogenic enhancers. *DUX4* is composed of 3 exons, exons 1 and 2 are present in each D4Z4 repeat, but exon 3 is located outside of the repeats. Three types of exon 3 have been described: exons 3a and 3b are transcribed from the 4A161L allele (dashed line) and exon 3 from 4A161S allele (plain line). Exon 3 carries the polyadenylation signal. Five *DUX4* isoforms have been characterized. The four leading to the full-length protein (DUX4-fl) are pathogenic, whereas the one leading to a truncated protein (DUX4-s) is non-pathogenic.

### 3.1. D4Z4 Epigenetic Modification

Because it is well known that epigenetic modifications play a significant role in gene regulation in normal and pathological environments, several studies have evaluated whether or not the epigenetic disruption observed at the 4q35 locus could lead to the expression of *DUX4*. In 2012, Lemmers and colleagues reported that antisense nucleotide-mediated exon skipping of *SMCHD1* in normal human myoblasts led to *DUX4* expression [18]. Combined with the observation that families with FSHD2 present a haploinsufficiency of SMCHD1 and a hypomethylation of the D4Z4 array [18], a link between epigenetic modifications and *DUX4* expression was established. Since then, several articles have reinforced the idea of an epigenetic regulation of DUX4 expression. The consequences of *SMCHD1* expression level on *DUX4* expression were particularly studied, and it was shown that SMCHD1 levels participate in *DUX4* expression in muscle cells. Indeed, depletion of *SMCHD1* in FSHD1 myoblasts increased *DUX4* expression [26] whereas its ectopic overexpression resulted in DUX4 silencing in FSHD1 and FSHD2 myotubes [27]. This is consistent with the fact that *DUX4* expression is increased during muscle differentiation, which correlates with decreased SMCHD1 protein levels at D4Z4 [27]. Moreover, the interaction of SMCHD1 with the chromatin is facilitated by the ligand-dependent nuclear receptor-interacting factor 1 (LRIF1), which binds to the D4Z4 repeat [28]. Interestingly, mutations in *LRIF1* lead to chromatin relaxation and *DUX4* derepression [28], and knockdown of the *LRIF1* long isoform in control myoblasts using siRNA results in the expression of DUX4 [28]. *DUX4* expression in myoblasts was also observed after decreased binding of SMCHD1 to D4Z4 caused by the inhibition of H3K9me3 (repressive mark associated with heterochromatin formation) using drugs [29]. Finally, a recent study has also shown that *DUX4* is expressed in myocytes obtained from patients presenting a 18p hemizygosity with a decreased of *SMCHD1* mRNA [30]. Altogether, these studies suggest a link between *SMCHD1*-mediated epigenetic modifications and DUX4 expression.

Multiple other lines of evidence show a role of epigenetics in DUX4 expression: (i) MyoD-converted fibroblasts isolated from FSHD2 patients carrying a mutation in the *DNA methyltransferase 3B* (*DNMT3B*) gene express DUX4, suggesting a D4Z4 derepression associated with DUX4 expression [19]. (ii) Several epigenetic pathways such as *ASH1L*, *BRD2*, *KDM4C*, and *SMARC5* were found to regulate DUX4 expression in primary FSHD cells after independent knockdown of multiple chromatin regulators [31]. (iii) Human chromosome 4/CHO hybrid cells treated with 5′-aza-2′deoxycytidine (AZA, a cytosine analogue that is incorporated into DNA during DNA replication) and/or trichostatin A (TSA, which inhibits class I and II histone deacetylases) led to *DUX4* expression [32,33]. (iv) Two D4Z4 factors, nucleosome remodelling deacetylase (NuRD) and chromatin assembly gactor 1 (CAF-1) were identified as DUX4 repressors in human skeletal muscle cells using RNA-guided Cas9 nuclease from the microbial clustered regularly interspaced short palindromic repeats (CRISPR/Cas9) engineered chromatin immunoprecipitation (enChIP) locus-specific proteomics to characterize D4Z4-associated proteins [34]. (v) Hemizygous transgenic mice carrying either a 2.5 or 12.5 D4Z4 repeat showed a chromatin relaxation of the D4Z4 repeats in D4Z4-2.5 mice compared to D4Z4-12.5 mice, associated with *DUX4* expression in the D4Z4-2.5 mouse [35].

Altogether, these studies strongly suggest that chromatin relaxation results in inappropriate DUX4 expression in skeletal muscle. However, regulation of *DUX4* expression may be different in other tissues or during development. Indeed, *DUX4* is expressed in early cleavage-stage embryos whereas a high methylation level is found at D4Z4 in pluripotent cells in both FSHD1 and controls [4,36], which goes against a link between D4Z4 hypomethylation and *DUX4* expression.

### 3.2. Chromatin Conformation

D4Z4 chromatin structure was also associated with *DUX4* expression/repression in muscle. Indeed, the 3D organization of chromatin modulates major biological processes including transcription. In regard of the link between DUX4 expression and chromatin conformation, it was proposed that, as a single repeat, D4Z4 behaves as a CCCTC-binding factor (CTCF) insulator interfering with enhancer–promoter communication [37]. However, both its CTCF binding and insulation properties are suppressed upon multimerization of D4Z4 units, suggesting that FSHD could result from an inappropriate insulation mechanism and a CTCF-gain of function [37]. Because CTCF can mediate transcriptional regulation by creating accessible or inaccessible loops of chromatin at specific sites, the involvement of CTCF in *DUX4* expression was proposed [38]. In this study, the authors found CTCF to be more readily associated with transcriptionally silent arrays, suggesting a role of CTCF in repressing *DUX4* transcription.

D4Z4 was also described as an insulator shielding from telomeric position effect (TPE). Indeed, telomeres can regulate gene expression by trapping adjacent heterochromatin. Using isogenic clones with different telomere lengths, it was demonstrated that telomere shortening led to *DUX4* expression [39]. The likely mechanism is that the epigenetic landscape is altered during telomere shortening resulting in decreased heterochromatin at 4q35 [40,41].

Interestingly, whereas the epigenetic modifications observed in FSHD patients at the D4Z4 array are not restricted to the muscle tissue [42,43,44], *DUX4* mRNA was found mainly in the skeletal muscle, testis, and thymus [5,45]. Two enhancers upstream of the D4Z4 that upregulate DUX4 expression in skeletal myocytes but not in fibroblasts were described [46]. Importantly, these enhancers participate in *DUX4* expression only when the *DUX4* promoter is hypomethylated. However, the exact role of these enhancers in FSHD onset may be questioned as two FSHD1 patients have been identified with large deletions encompassing this chromosomal region [47]. Moreover, meiotic rearrangements between chromosomes 4 and 10 [14,48] go against a central role of other regions of chromosome 4 in *DUX4* expression.

### 3.3. Regulatory Proteins of DUX4 Expression

Transcriptional regulation of DUX4 expression may be also controlled by gene regulatory proteins that interact with the DUX4 promoter, and one study identified Poly(ADP-Ribose) Polymerase 1 (*PARP1*) using a DNA pull-down assay coupled with mass spectrometry and chromatin immunoprecipitation [49].

Several inhibitors of *DUX4* have been published, suggesting that the target inhibitors may play a role in *DUX4* expression. It was shown that activation of the Wnt/β-catenin signalling reduced *DUX4* expression whereas knockdown of Wnt/β-catenin signalling pathway components activates DUX4 [50]. The mechanism of *DUX4* regulation by Wnt/β-catenin is likely independent of direct binding of β-catenin at D4Z4. Bromodomain and extra-terminal (BET)- and β2 adrenergic receptor-mediated pathways were also associated with DUX4 expression regulation [51]. Using BET inhibitors (BETi) targeting all proteins of the BET family, *DUX4* and DUX4 target candidates were silenced in primary FSHD muscle cells [51]. The research team suggested that BETi efficiently repressed *DUX4* transcription by lysine deacetylation but not DNA methylation. Similarly, β2 adrenergic receptor agonists activate signalling pathways known to induce chromatin remodelling. *DUX4* and DUX4 target candidates’ expression were both repressed following treatment with β2 adrenergic receptor agonists, suggesting the role of BET and β2 adrenergic receptor signalling pathways in DUX4 expression in FSHD patients [51]. Since then, the importance of the β2 adrenergic receptor has been confirmed in additional studies [52], and downstream pathways have been the centre of attention in order to identify therapeutic targets. P38 mitogen-activated protein kinase is activated by the β2 adrenergic receptor signalling pathway [53]. In FSHD muscle cells or in a xenograft model of FSHD, pharmaceutical or siRNA-mediated inhibition of p38 induced a reduction of *DUX4* mRNA levels [54]. This suggests that β2 adrenergic receptor agonist-mediated *DUX4* expression is a consequence of p38 kinase activation. Phosphodiesterases, or PDEs, which are responsible for regulation of available cAMP in the cell, were identified as *DUX4* expression regulators [52] by reducing expression levels of both DUX4 and its target genes *ZSCAN4* and *TRIM43*. β2 adrenergic receptor and PDEs are both implicated in cAMP-mediated signalling that further regulates protein kinase A (PKA) signalling pathways. Both cell-permeable cAMP and catalytic active PKA were sufficient to reduce *DUX4* expression and *ZSCAN4* and *TRIM43* mRNA levels [52] in primary FSHD patients’ muscle cells. The authors suggested that β2 adrenergic agonists and PDE inhibitors mediated a c-AMP and PKA-mediated repression of DUX4 gene expression in FSHD muscle cells. However, downstream effectors of cAMP also include PKA-independent pathways, and the results from Campbell et al. suggest a PKA-independent mediated repression of DUX4 [51]. Later, p38α and p38β MAPK inhibitors were identified as suppressors of *DUX4* mRNA transcription in myotubes and in a xenograft model of FSHD [54], suggesting a positive regulation of *DUX4* transcription by both p38α and p38β.

## 4. *DUX4* mRNA

### 4.1. DUX4 Transcription

The presence of a large ORF encompassing 2 homeoboxes in each D4Z4 repeat was first described in 1995 [55], but the identification of the DUX4 gene occurred in 1999 by the Belayew group [7]. This group also identified the DUX4 promoter with a variant of TATAA box (TACAA) [7]. The final demonstration that D4Z4 contains a functional DUX4 transcriptional unit leading to the *DUX4* transcription was made few years later after cloning of the D4Z4 region into a promoter-less vector and transfection into myoblasts [56]. 5′ Rapid amplification of cDNA ends (RACE) PCR lead to the identification of the 5′untranslated region (UTR) composed of 97–187 nt [56]. The polyadenylation site was described after 3′ RACE PCR on total RNA extracted from C2C12 mouse myoblasts transfected with a 13.5 kb genomic fragment of a patient with two D4Z4 repeats [57]: It is the ATTAAA hexanucleotide sequence (12852–12858 in GenBank accession no. AF117653).

The *DUX4* mRNA found in the muscle tissue is composed of 3 exons, with the *DUX4* ORF being entirely within exon 1. Importantly, exons 1 and 2 are present in the D4Z4 repeats but not exon 3, which is located in region called pLAM. Notably, the pLAM region is not present on the 4qB haplotype that is classified as non-pathogenic [22,58]. This leads to the hypothesis that DUX4 would only be transcribed for the most telomeric repeat because only this one would give rise to a polyadenylated DUX4. The role of this region in DUX4 expression and stability was highlighted by the report of individuals with a genomic rearrangement between chromosome 4q and 10q. Indeed, the subtelomeric part of these 2 chromosomes is highly homologous and, importantly, chromosome 10 does not carry the ATTAAA poly(A) signal found in chromosome 4, but an ATCAAA sequence that is not known to be a poly(A) signal [14]. Meiotic rearrangements between chromosomes 4 and 10 generated a short hybrid structure on 4qA where the pLAM sequence was conserved but immediately proximal to a 1.5 D4Z4 repeat coming from chromosome 10, resulting in disease presentation. Transfection experiments with genomic D4Z4 constructs derived from permissive or non-permissive chromosomes or in which the poly(A) signals from non-permissive chromosomes are replaced by those from permissive chromosomes established the importance of this poly(A)signal in the stabilization of *DUX4* [14].

Two different *DUX4* mRNAs, resulting from the inclusion or exclusion of an alternatively spliced intron of 136 bp located in the 3′UTR part of mRNA have been described [57]. The two *DUX4* mRNAs have also spliced out a 345 bp intron also located in the 3′UTR region [57]. These two *DUX4* mRNAs were later renamed DUX4-full length (DUX4-fl) [5]. Recently, other *DUX4* mRNAs have been characterized from a common variant of the most prevalent FSHD-permissive haplotype 4A161 (containing an SSLP of 161 nt and the distal 4qA variant [59]). These two variants present a 1.6 kb size difference of the most distal D4Z4 units [60]. Two *DUX4* mRNAs are transcribed from this long allele using 2 alternative 3′ splice sites, leading to either the DUX4-fl 161La or Lb transcripts (Figure 1) (GenBank accession numbers MF693913 and KQ983258.1). The three pathogenic DUX4-fls share the pLAM sequence containing the DUX4 poly(A) and lead to the same DUX4 protein. There is no link between disease severity and transcript variants [60].

### 4.2. DUX4 Isoforms

DUX4 transcription from the last D4Z4 repeat results in at least 5 different mRNAs, the 4 *DUX4-fls* described above, code for the same protein but differ by an altered splicing of intron 1 in the 3′UTR and by the use 2 alternative 3′ splice sites leading to different types of exon 3. The fifth *DUX4* transcript corresponds to a short version of DUX4 (DUX4-s), in which an alternative donor splice site located in first exon is used [24], leading to a truncated form of DUX4, lacking the C-terminal part of the protein containing the transactivation domain [61] and acting as a dominant negative [25]. *DUX4-fl* isoforms are mainly found in myotubes and muscles biopsies isolated from FSHD patients, whereas *DUX4-s* can be found in both control individuals and FSHD patients [5,62]. DUX4-fl expression increases in myotubes [5,62,63]. An isoform switch may be possible, since it was shown in iPS cells derived from control fibroblasts that *DUX4*-fl is expressed in undifferentiated cells but can switch to *DUX4*-s in embryoid bodies [5]. *DUX4-fl* mRNA is expressed in muscles during development, as both isoforms are found in foetal muscle biopsies and cells derived from foetal muscle [64,65].

Interestingly, *DUX4* mRNA is also found in human testes at a level 100-fold higher compared to FSHD muscle biopsies [5] but does not seem to be toxic. 3′ RACE PCR analysis revealed that both chromosomes 4 and 10 were used for *DUX4* transcription, despite the absence of a permissive poly(A) signal on chromosome 10. Chromosome 10 and some 4qA transcripts use an alternative poly(A) located in exon 7. Surprisingly, *DUX4* transcripts were also found from the 4qB allele, but the poly(A) still need to be identified. Exons 3 and 7 are excluded since they are not present in the 4qB allele. Non-canonical poly(A) signals may be also used in some circumstances, as observed in the presence of antisense oligonucleotides targeting the poly(A) signal [66]. The use of alternative poly(A) signals could also explain the normal embryogenesis observed in individuals carrying non-permissive 4q alleles. Consistent with this hypothesis, studies have also shown that alternative polyadenylation pattern varies among cell types [67] and during embryonic development [68].

## 5. DUX4 Low Abundancy and Stochastic Expression

*DUX4* mRNA is found at a very low level in both biopsies and muscle cells from both FSHD1 and FSHD2 patients. This low abundance could reflect a uniform low level in all nuclei or a high expression in a limited number of nuclei. By pooling a different number of nuclei and after assessment of the presence of *DUX4-fl* by PCR, it has been estimated that about 1 in 1000 FSHD nuclei are positive for *DUX4* mRNA [5]. The question is how could a gene expressed at such low levels be so toxic? The presence of the endogenous DUX4 protein in consecutive myotube nuclei, forming an intensity gradient, suggested a spreading of the protein within the myotubes [69]. This hypothesis was confirmed by co-culture experiments between FSHD myoblasts and murine C2C12 myoblasts. Whereas *DUX4* is transcribed in human nuclei only, the protein was found in both human and murine nuclei showing the spreading of the DUX4 protein [70]. The sporadic and asynchronous burst of expression of DUX4 was confirmed using a *DUX4*-activated reporter [71].

## 6. Conclusions

During the past decade, our knowledge about FSHD onset considerably improved. Several genetic and epigenetic defects have been clearly identified that cause FSHD, all leading to the aberrant expression of the DUX4 transcription factor. Once expressed, DUX4 triggers a cascade of events that ultimately converge to cell death and impair muscle development and repair (for review see [8]). After years of controversy, DUX4 is now seen as one of most important players in FSHD onset and progression. Some areas remain unelucidated, such as the non-toxic expression of DUX4 during embryogenesis [2,4] or the different splicings observed in the testis [5]: Are they due to a difference between pathogenic and healthy environment or are they tissue-specific?

Multiple studies have deciphered the expression of DUX4 in skeletal muscle and demonstrated that chromatin conformation, DNA methylation and histone modification, myogenic enhancer, and regulatory proteins are involved in the regulation of its expression. Moreover, some other repressor proteins or lncRNA that are associated with the D4Z4 repeat may also play a role [32,72].

Several laboratories are developing therapeutic approaches targeting DUX4 by either blocking *DUX4* mRNA synthesis [31,51,52,54], targeting *DUX4* mRNA using antisense oligonucleotides [66,73,74,75,76], or targeting the DUX4 protein or its downstream consequences [77,78,79]. One phase 2 clinical trial (NCT04003974) aiming at inhibiting or reducing its expression in skeletal muscle is already on-going and may enable a better understanding of the role of DUX4 in the pathophysiology of FSHD.
